# NHS-Reimbursed Cannabis Flowers for Cancer Palliative Care and the Management of Chemotherapy-Induced Nausea and Vomiting: An Autobiographical Case Report

**DOI:** 10.7759/cureus.61791

**Published:** 2024-06-06

**Authors:** Michael Roberts, Matthew R D Brown, Guillermo Moreno-Sanz

**Affiliations:** 1 Research, Independent Researcher, Southampton, GBR; 2 Pain Medicine, The Royal Marsden Hospital, London, GBR; 3 Pharmacology, Khiron Europe, Madrid, ESP; 4 Pharmacology, Zerenia Clinics, London, GBR

**Keywords:** nabilone, individual funding request, nhs, tetrahydrocannabinol (thc), cannabis flowers, chemotherapy-induced nausea and vomiting (cinv)

## Abstract

Chemotherapy-induced nausea and vomiting (CINV) is a debilitating side effect of cancer treatment, affecting many patients. Cannabinoid agonists, such as nabilone and Δ9-tetrahydrocannabinol (THC), the main psychoactive component of *Cannabis sativa *L., have shown efficacy as antiemetics.

Here, we report the case of Michael Roberts (MR), who we believe is the first British patient reimbursed by the National Health Service (NHS) England for the cost of medicinal cannabis flowers to manage CINV. Medical data were obtained from NHS records and individual funding request (IFR) forms. Patient-reported outcome measures (PROMs) were collected using validated questionnaires as part of the standard of care at the specialized private clinics where the prescription of medicinal cannabis was initiated.

The patient presented with rectosigmoid adenocarcinoma with lung metastases. He received FOLFIRI (folinic acid, fluorouracil, and irinotecan) chemotherapy and underwent an emergency Hartmann's procedure with subsequent second-line FOLFOX (folinic acid, fluorouracil, and oxaliplatin) chemotherapy and lung ablation. MR reported severe nausea and vomiting associated with the initial FOLFIRI treatment. Antiemetics metoclopramide and aprepitant demonstrated moderated efficacy. Antiemetics ondansetron, levomepromazine, and nabilone were associated with intolerable side effects. Inhalation of THC-predominant cannabis flowers in association with standard medication improved CINV, anxiety, sleep quality, appetite, overall mood, and quality of life.

Our results add to the available evidence suggesting that medicinal cannabis flowers may offer valuable support in cancer palliative care integrated with standard-of-care oncology treatment. The successful individual funding request in this case demonstrates a pathway for other patients to gain access to these treatments, advocating for broader awareness and integration of cannabis-based medicinal products in national healthcare services.

## Introduction

Chemotherapy-induced nausea and vomiting (CINV) is a distressing and often debilitating side effect of cancer treatment. It affects a significant proportion of cancer patients, with studies suggesting it impacts up to 40% of those undergoing chemotherapy [1]. Traditional antiemetic medications, while effective for many, are not always successful in managing CINV and may also produce side effects, such as headaches and constipation, which further compromise patients’ quality of life [2,3]. With the legalization of cannabis-based medicinal products (CBMPs) in the UK, there has been growing interest in their potential role in managing CINV and other cancer-related symptoms. Cannabinoid agonists nabilone and Δ9-tetrahydrocannabinol (THC), alone or in combination with standard medication, have shown promise as effective antiemetics and have been associated with a range of other therapeutic benefits [4].

This case report focuses on the experience of Michael Roberts (MR), who we believe is the first NHS patient to be reimbursed for the use of medicinal cannabis flowers to manage CINV. The primary objective of this case study is to explore the efficacy of THC-predominant cannabis flowers in rapidly controlling CINV and potentially alleviating other cancer-related symptoms such as anxiety, loss of appetite, and sleep deterioration.

## Case presentation

In May 2021, MR, a 42-year-old male previously fit and well mathematics teacher, was diagnosed with metastatic MMR proficient RAS mutant BRAF wild-type rectosigmoid adenocarcinoma with multiple lung metastases. He commenced palliative FOLFIRI chemotherapy. In August 2021, MR was admitted as an emergency with bowel perforation after two cycles of chemotherapy. A Hartmann's procedure was performed, removing the primary tumor (ypT3 N2a) and controlling sepsis. FOLFIRI chemotherapy was resumed in December 2021 and 10 additional cycles were completed by May 2022. Progression of lung metastasis necessitated the start of second-line chemotherapy with FOLFOX (folinic acid, fluorouracil, and oxaliplatin) in September 2022. After completing FOLFOX chemotherapy in February 2023, MR underwent radiofrequency lung ablation.

Management of CINV with THC-predominant flowers

MR reported severe nausea and vomiting associated with the initial FOLFIRI treatment. He was prescribed metoclopramide and aprepitant, which offered limited benefit, and ondansetron, which resulted in constipation and was discontinued after his Hartmann's procedure. After resuming FOLFIRI chemotherapy, MR sought a private consultation and in May 2022, he obtained his first private prescription for THC-predominant cannabis flowers for inhalation. In the UK, the majority of CBMPs are unlicensed medicines or “specials” and while they can be prescribed by clinicians complying with strict guidance from NHS England, the Medicines and Healthcare Products Regulatory Agency (MHRA), and the General Medical Council (GMC), funding of these medications is challenging due to the lack of National Institute for Health and Care Excellence (NICE) formal guidelines. Unlicensed CBMPs most frequently prescribed in the UK are dried flowers for inhalation and oral extracts, and are usually identified by the relative composition of their active ingredients, i.e., THC and cannabidiol (CBD), and classified by chemotype as follows: chemotype-1 (THC-predominant, THC>>>CBD); chemotype-2 (balanced, THC≈CBD); and chemotype-3 (CBD-predominant, THC<<<CBD) [5].

After initiating the second-line FOLFOX chemotherapy in September 2022, MR was prescribed levomepromazine, which resulted in severe drowsiness, and nabilone, which did not help with CINV. To provide a comparative assessment of the efficacy of the interventions tested, MR retrospectively completed the revised Edmonton Symptom Assessment Scale (ESASr) in May 2023 [6]. Results are expressed in Table 1 and show that inhalation of cannabis flowers together with metoclopramide and aprepitant was the most successful combination for controlling CINV.

**Table 1:** Results from the ESASr questionnaire filled by Michael Roberts in May 2023. The revised Edmonton Symptom Assessment Scale (ESASr) questionnaire assigns a value on a scale from 0 to 10 to each symptom associated with cancer palliative care, with "0" being the least possible and "10" being the worst possible symptomatic impact [6]. The patient reported maximal control over chemotherapy-induced nausea and vomiting (CINV) with Δ9-tetrahydrocannabinol (THC)-predominant cannabis flowers as an adjunct to standard antiemetics. On the contrary, nabilone was the least effective treatment option for controlling nausea and lack of appetite. Additionally, treatment with cannabis-based medicinal products (CBMPs) was associated with improved self-reported anxiety, depression, and overall physical well-being.

Symptoms	No treatment	Metoclopramide + aprepitant	Metoclopramide + aprepitant + nabilone	Metoclopramide + aprepitant + THC-predominant cannabis flowers
Pain	0	0	0	0
Tiredness	9	9	9	2
Drowsiness	6	6	8	2
Nausea	9	7	10	0
Lack of appetite	9	8	10	0
Shortness of breath	8	8	8	3
Depression	9	8	9	1
Anxiety	9	8	9	1
Well-being	9	8	9	3

Funding of THC-predominant cannabis flowers by the NHS

In July 2022, MR requested his general practitioner to submit an individual funding request (IFR) to the NHS South, Central, and West Commissioning and Support Unit to cover the cost associated with his medicinal cannabis flowers. This amounted to £388 per month for the 24 months his oncologist had estimated his prognosis to be. In October 2022, the panel denied his petition considering by mistake that MR was requesting funding for the use of Sativex. Sativex is a CBMP authorized for the treatment of spasticity associated with multiple sclerosis, and the funding panel argued that Sativex was not approved for the indication of CINV. In November 2022, the IFR was resubmitted, clarifying that the application was for medicinal cannabis flowers, an unlicensed CBMP. The panel then denied this second request because MR had not exhausted other authorized options like nabilone.

In March 2023, after trialing nabilone and finding it of minimal efficacy, MR’s general practitioner resubmitted the IFR, emphasizing that the use of cannabis flowers together with prescription antiemetics allowed the patient to complete his course of FOLFOX chemotherapy. This was further corroborated by MR’s oncologist in a letter to The Shirley Health Partnership supporting MR’s IFR. On April 20th, 2023, the funding panel met and agreed to fund MR’s request. The panel also agreed to fund the cost of a certified medical device, an herbal vaporizer, for the administration of medicinal cannabis flowers in compliance with current UK legislation set forth by the Home Office in 2018, which prohibits smoking of CBMP.

Palliative use of THC-predominant cannabis flowers in advanced or metastatic cancer

Besides offering control of CINV, the use of CBMP allowed MR to tolerate other symptoms associated with his cancer and its treatment. In MR’s words, “using medical cannabis transforms me from an anxiety-ridden, vomiting, fatigued wreck into an active family participant, playing with my kids, chatting with my wife and friends, cooking meals, and cleaning the house. It enables me to make the most of the time I have left, whether I'm on chemo or recovering from it. I want to use medical cannabis to have the best possible quality of life for my remaining years.” Patient-reported outcome measures were collected at the onset of treatment and at two- and four-month follow-ups as part of the standard of care received at the private clinics where MR obtained his prescription for CBMP. Validated questionnaires, including the General Anxiety Disorder-7 (GAD-7) and the single-item Sleep Quality Scale (SQS), were used [7,8]. Results reported in Table 2 show improvements in anxiety and quality of sleep associated with the addition of CBMP to the standard of care management. These improvements mirror what was reported by the patient in the ESASr following the usage of chemotype-1 cannabis flowers, as shown in Table 1. Coincidentally, the patient was able to discontinue the use of prescription drugs, such as diazepam and zopiclone, and to reduce the dose of fluoxetine. Administration of cannabis flowers was performed “on demand” and amounted typically to one gram per day. Varieties of​​​ ​​​​*Cannabis sativa* L. used were of chemotype-1 (THC-predominant) and contained an average of 20% of THC and less than 1% of CBD in dry weight. Individual dosing size was 0.2 grams, which amounts to a maximum of 40 mg of THC per administration. However, the actual absorption of THC largely depends on the number of inhalations and the temperature used [5,9].

**Table 2:** PROMs questionnaires filled by Michael Roberts before CBMP prescription and at the two- and four-month follow-ups. Analysis of clinical outcomes reported in the GAD-7 (scoring from 0 being no anxiety to 21 being worst anxiety), and SQS (scoring from 0 being terrible sleep to 10 being excellent sleep) questionnaires indicates improvements in clinical anxiety and quality of sleep associated with the controlled inhalation of THC-predominant cannabis flowers [7,8]. PROMs: patient-reported outcome measures; CBMP: cannabis-based medicinal products; GAD-7: General Anxiety Disorder-7; SQS: Sleep Quality Scale; THC: Δ9-tetrahydrocannabinol.

	February 3^rd^, 2022	April 1^st^, 2022	May 31^st^, 2022
Questionnaire	Initial consultation	2-month follow-up	4-month follow-up
GAD-7	11	7	0
SQS	3	4	8

## Discussion

The clinical efficacy of THC to mitigate CINV was first established in 1985 when the FDA authorized the commercialization of dronabinol (oral THC) for this indication. Controlled vaporization of chemotype-1 cannabis flowers allows for the rapid absorption of THC, bypassing first-pass metabolism, which offers the patient better control over symptoms compared with the slow oral kinetics of dronabinol [5]. THC is believed to exert its antiemetic properties through the activation of both central and peripheral CB1 receptors and serotonergic 5-HT3 receptors in the dorsal vagal complex (DVC) [3,10]. In the present case study, we report the efficacy of the controlled inhalation of THC-predominant cannabis flowers by means of a medical device for the rapid control of acute and delayed CINV in a patient with advanced metastatic cancer.

Administration of CBMP also improved concomitant symptoms of anxiety, depression, and sleep disturbances as reported by validated questionnaires. These improvements allowed the patient to discontinue other medications such as ondansetron, levomepromazine, diazepam, and zopiclone. This report also highlights the low level of awareness amongst the medical community about the therapeutical potential of CBMP to palliate CINV and the difficulties for UK patients to access this class of medication despite their use being permitted since 2018. The NHS maintains a patient registry for the collection of observational information for patients who are taking licensed and unlicensed CBPMs under prescription (https://www.england.nhs.uk/long-read/cannabis-based-products-for-medicinal-use-patient-registry/). However, the registry remains virtually unused due to the restricted access to these medications by NHS patients.

Nevertheless, to the best of our knowledge, MR was able to become the first patient to be granted funding to cover the cost of cannabis flowers likely due to two key aspects of his case: (i) CINV is, together with multiple sclerosis and certain pediatric epileptic syndromes, an indication approved for the prescription of an authorized CBMP (nabilone), and (ii) his IFR was for a limited duration considering his, unfortunately, short prognosis. However, this case adds to the current evidence suggesting the administration of THC via controlled inhalation of cannabis flowers may offer valuable support in cancer management within national healthcare services [11]. Further observational research is warranted to explore the full potential and safety profile of medicinal cannabis in cancer management and to inform evidence-based treatment guidelines.

## Conclusions

This case report highlights the potential of THC-predominant cannabis flowers in the management of CINV in a cancer patient, marking a significant step in palliative cancer care. Michael Roberts, who we believe is the first NHS patient reimbursed for medicinal cannabis flowers, experienced substantial relief from CINV, alongside improvements in pain, anxiety, sleep, appetite, and overall quality of life. His case underscores the therapeutic benefits of controlled inhalation of cannabis flowers, particularly in patients unresponsive to conventional antiemetics. This report brings further attention to the challenges faced by patients in accessing CBMPs within the NHS, despite their legalization and acknowledged potential in symptom management. The successful individual funding request in this case demonstrates a pathway for other patients to gain access to these treatments, advocating for broader awareness and integration of CBMPs in national healthcare systems.

Further research and observational studies are needed to expand the evidence base, guide clinical practice, and potentially revise national guidelines to better support the integration of medicinal cannabis in the standard oncology care protocols. This case adds valuable insight into the practical application and benefits of CBMPs, suggesting a promising adjunctive role in the palliative care of patients with metastatic or advanced cancer.
